# Radiotherapy for prostate cancer: DISCERN quality assessment of patient-oriented websites in 2018

**DOI:** 10.1186/s12894-019-0474-4

**Published:** 2019-05-28

**Authors:** S. Janssen, F. B. Fahlbusch, L. Käsmann, D. Rades, D. Vordermark

**Affiliations:** 1 Department of Radiation Oncology, University of Luebeck, Luebeck, Germany; 2 Department of Pediatrics and Adolescent Medicine, Friedrich-Alexander-University Erlangen-Nuernberg, Erlangen, Germany; 3 Department of Radiation Oncology, University Hospital Halle (Saale), Halle, Germany; 4 Medical Practice for Radiotherapy and Radiation Oncology, Hannover, Germany

**Keywords:** Prostate cancer, Radiotherapy, Internet, Health-related information, Online information

## Abstract

**Background:**

Prostate cancer is the most commonly diagnosed cancer in men. Radiotherapy represents one major treatment option in different therapeutic settings. As patients increasingly rely on internet-based medical information, we examined the quality of information on radiotherapy and prostate cancer in websites used by laypersons.

**Methods:**

An Internet search from a patients` perspective was carried out using different search engines (Google, Yahoo and Bing, search terms: “prostate cancer” and “radiotherapy”). The quality of search results was analyzed with regard to the DISCERN score, HON code certification, the JAMA criteria and the ALEXA traffic rank.

**Results:**

In general, websites were of good quality. The highest quality was found for websites operated by charity organizations. No significant differences in results obtained via the above-mentioned tools were seen for the examined search engines, but Google revealed the most stable search results in terms of temporal changes.

**Conclusion:**

Patients with prostate cancer can sufficiently inform themselves on general treatment options including radiotherapy on websites directed at laypersons. However, no simple strategy could identify high quality websites in general. For treating physicians, it is important to support patients in interpreting and ranking the vast quantity of information.

**Electronic supplementary material:**

The online version of this article (10.1186/s12894-019-0474-4) contains supplementary material, which is available to authorized users.

## Background

Prostate cancer is the most commonly diagnosed cancer in the male population worldwide [1]. Radiotherapy for localized prostate cancer remains one of the mainstays among treatment approaches. Other fields of application are biochemical recurrences after prostatectomy, postoperative radiotherapy in high-risk situations and palliative settings e.g. painful bone metastases [2, 3]. As most patients do not have personal experiences with radiotherapy, a lack of information on risks and benefits is immanent. A recent Canadian survey revealed that more than 90% of men with newly diagnosed prostate cancer prefer to either play an active or collaborative role in treatment decision-making [4]. Thus, gaining valid medical information before initiating treatment seems essential for these patients to improve their decision-making.

Today, a significant number of patients with prostate cancer [5, 6] and radiation oncology patients [7, 8] utilize the Internet to obtain medical information. For cancer patients in general, Castleton et al. reported Internet utilization in approximately two thirds of patients [9]. However, the quality of online information for patients with prostate cancer is variable [6, 10–12]. For instance, Ilic et al. found the quality of websites on prostate cancer screening to be highly variable but mostly of poor standard [10]. Similarly, others found shortcomings in quality and accuracy of Internet health information for the special topic of prostate cancer and proton therapy [11]. Noteworthy, Shaverdian et al. showed that 39% of patients who selected the Internet as their primary information source reported their actual treatment experience to be worse than expected. Patients who had initially gathered medical information by consulting their urologists, radiation oncologist or even other patients reported a significantly better treatment experience [6]. On the other hand, a recently published analysis revealed a high rating in quality, accessibility and usability of websites on prostate cancer in general [12].

To our knowledge, there is no current analysis of consumer health information on the Internet for patients with prostate cancer in need of a radiation therapy. In order to close that gap, our goal was to evaluate websites with the focus on prostate cancer and radiotherapy.

## Methods

### Website identification and selection

A complete list of analyzed and excluded websites is given in Additional file 1 of the appendix. Identification and selection of websites (English language) took place on August 22nd 2017 (9.00 a.m. CET). The keywords “prostate cancer” and “radiotherapy” were entered in the search-engines Google.com, Bing.com and Yahoo.com to emulate real user experience. A second search was carried out on September 9th 2017 (9.00 a.m. CET) to reveal possible temporal dynamics. The first 20 websites were subjected to further evaluation as it is known that patients rarely browse through further sites when searching the web [12–14]. To simulate a laypersons view, we excluded websites from further analysis when complete access was restricted by password, the websites only included personal experiences (e.g. blogs, videos) or contained limited information on radiotherapy (< one paragraph), as described by others [15, 16]. In detail, websites were excluded based on the following criteria: advertisement only (*n* = 2), a focus on clinical trials (*n* = 4), scientific articles (*n* = 20), newspaper articles (n = 2), video (n = 2), denied access (n = 2) and PowerPoint presentation (*n* = 1). The vast majority of excluded websites was found using the search engines Bing.com and Yahoo.com. For the Google search, only one website met the above exclusion criteria.

After the subtraction of duplicate websites (i.e. repeated appearance in different search engines and time points) a total number of 49 websites was subjected to further evaluation (*n* = 39 first search, *n* = 10 second search). Apart from HON code, JAMA benchmark criteria, ALEXA rank and DISCERN score, the country of origin and website operator were recorded for each website additionally. The quality of websites was evaluated by two investigators (SJ, LK) independently using the validated tools described below. Discrepancies were discussed and a consensus was reached.

The DISCERN tool was originally developed and validated in 1998 at the University of Oxford, UK. The aim was to analyze written medical information with 15 questions in terms of reliability and details on treatment (score of 1–5 for each question) [17, 18]. Overall quality was additionally rated by a 16th question in the DISCERN Plus score. As described by Nghiem et al. the results were rated from “excellent” to “very poor” [19]. This methodology was used in a similar way in a previous published work of our study group [20]. The individual DISCERN items used in our study are visualized in Table 1. Following DISCERN scoring, the respective websites were further analyzed by using HON code [21] and JAMA [22] (tools, as outlined by us in detail earlier [20]. With the ALEXA traffic tool (https://www.alexa.com/) popularity and engagement characteristics of websites can be assessed. It is a measure of how often a website is frequented relative to all other sites on the web over the past three months.

**Table 1:** DISCERN Plus instrument (modified according to Borgmann et al.)

Question 1: Aim clear?	
Question 2: Aim achieved?	
Question 3: Relevant?	
Question 4: Sources clear?	
Question 5: Dates of sources?	
Question 6: Balanced and unbiased?	
Question 7: Additional information?	
Question 8: Areas of uncertainty?	
Question 9: Describe treatment?	
Question 10: Benefits of treatments?	
Question 11: Risks of treatment?	
Question 12: No treatment?	
Question 13: Quality of life?	
Question 14: Treatment choices?	
Question 15: Shared decision-making?	
Question 16: Overall quality?	

### Statistical analyses

We performed statistical analysis using GraphPad Prism 7 (GraphPad Software Inc., La Jolla, CA, USA) employing Spearman correlation, linear regression analysis and two-tailed non-parametric Wilcoxon-Mann-Whitney test for group comparisons as described in a previous published work of our study group in detail [20].

## Results

**Fig. 1:**
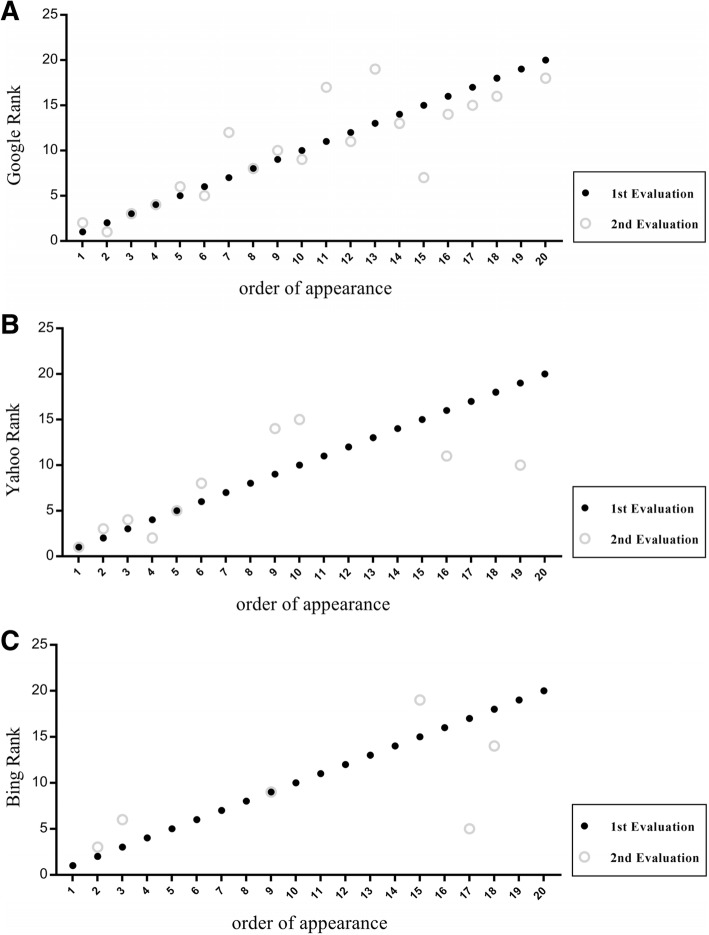
**a**-**c**: Google, Bing and yahoo search ranking at two different time points (black dots: august 22nd 2017, Open circles: September 9th 2017)

First, we analyzed the consistency of our data (i.e. exclusion rates, duplicate websites and temporal changes in search rank). Web search results obtained via Google seemed adequately directed at laypersons (one website excluded) and were highly consistent, as no duplicate results occurred and only a single new webpage was evident within the 20 hits in our second search after three weeks (Fig. 1A). In contrast, exclusion rates (*n* = 8 and 25) and the incidence of double hits (n = 8 and 10) and of novel search results/ranks (*n* = 10 and 14, Fig. 1B, C) were higher when Bing and Yahoo were employed, respectively.

**Fig. 2:**
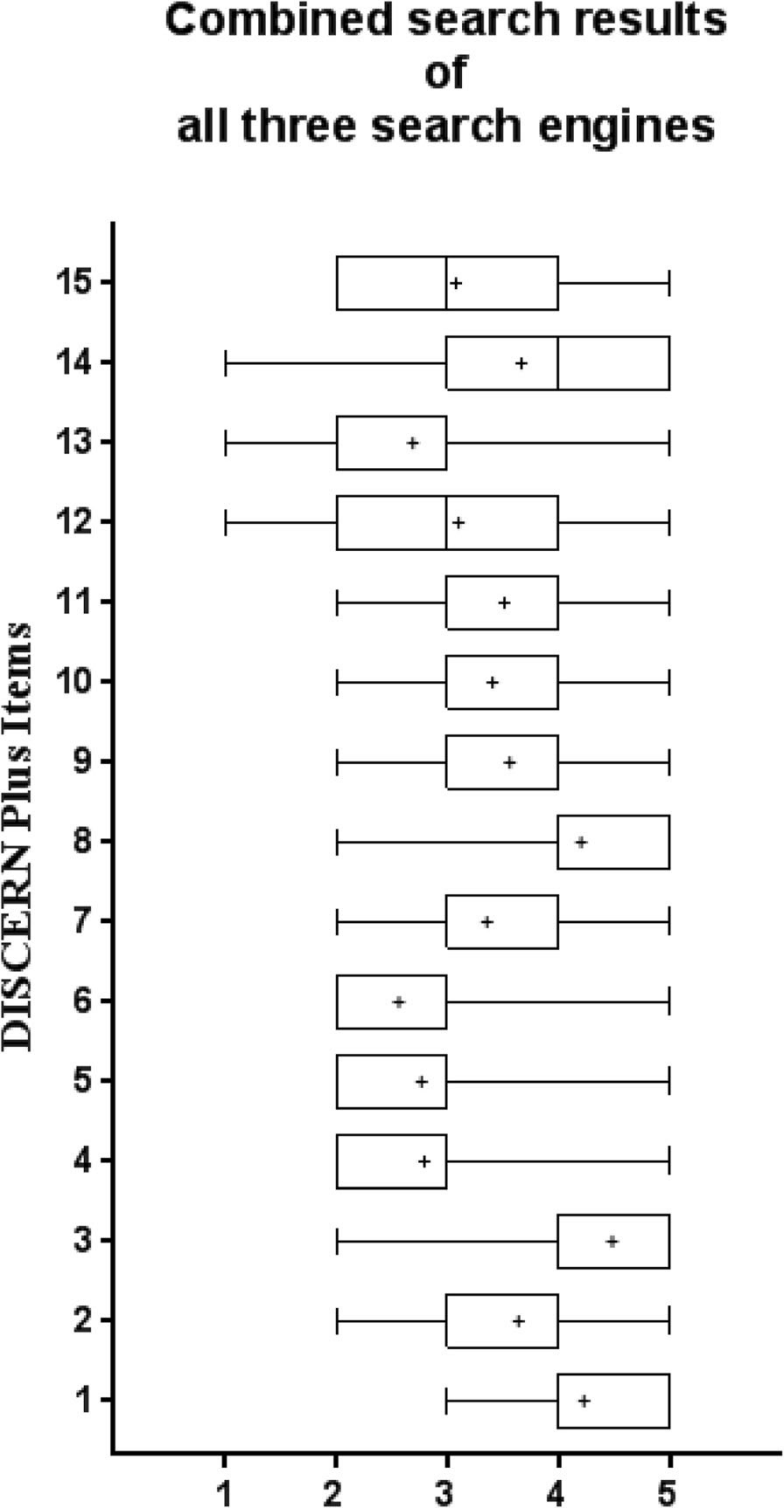
Combined (*n* = 39 from Google, Bing and Yahoo) box and whiskers (min to max) analysis of the individual DISCERN Plus score items of the first search (mean indicated by +)

**Fig. 3:**
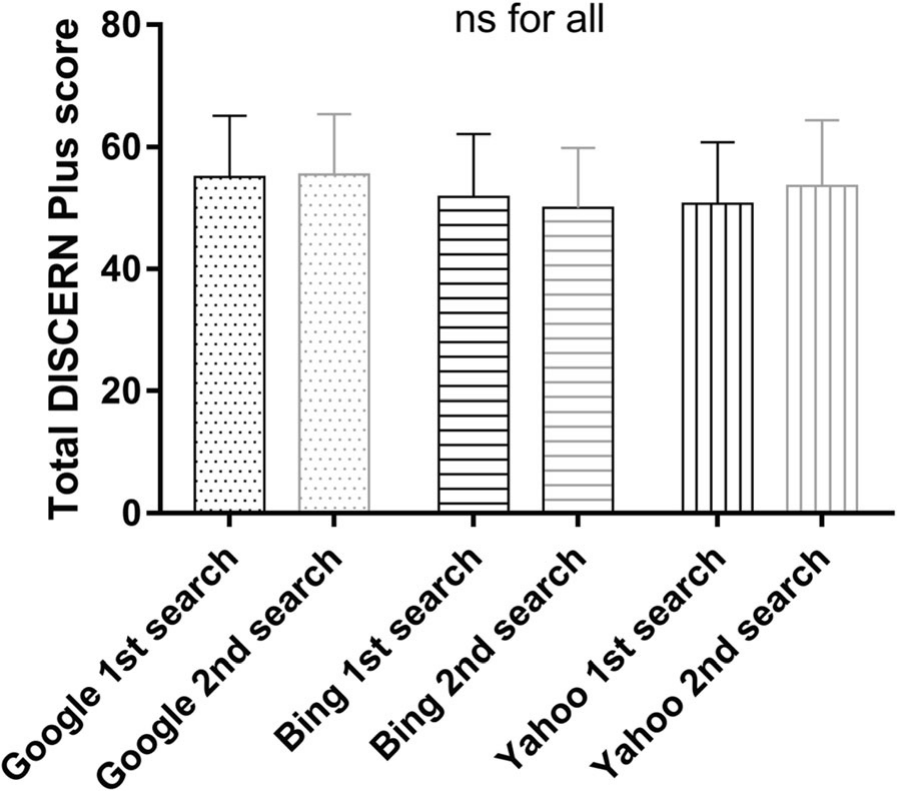
Mean total DISCERN score for the three search engines at two different time points

Overall, the quality of information on websites about prostate cancer and radiotherapy was good, with a mean DISCERN Plus score of 55.1 ± 10.0, 51.8 ± 10.3 and 50.7 ± 10.1 for Google, Yahoo and Bing, respectively. The scores ranged from a minimum of 34 to a maximum of 74. 13% of all websites were rated as excellent, 31, 48 and 8% as good, fair and poor, respectively. No website was rated as very poor. Figure 2 shows the item-based results of the DISCERN score for the first search results on August 22nd 2017 of all three search engines (*n* = 39 websites in total). The items with the lowest scores were related to website content concerning sources/references, up-to-dateness and quality of life issues (question 4–6 and 13, Table 1). The overall quality of websites (DISCERN Plus score) was neither dependent on the choice of search engines nor did we observe a significant temporal change in quality (Fig. 3). The main categories of websites retrieved from the first search were charity/NGO sites (46%), followed by sponsored medical news sites (28%), hospital/university sites (20%) and governmental sites (6%). Websites operated by charity organizations had significantly higher DISCERN Plus scores (mean score: 55.5 ± 9.3) compared to hospital sites (mean score: 47.3 ± 9.6, *p* < 0.042) and medical news sites (mean score: 46.1 ± 6.1, *p* < 0.009), respectively. Most websites originated in the USA (46%) followed by the UK (33%), Australia/New Zealand (15%) and Canada (6%). The JAMA benchmark criteria were fulfilled for all four sections in 13%, for three, two and one section(s) in 13, 31 and 40%, respectively. One website did not fulfill any of the JAMA criteria. Only 13% of all websites were HON code certified. The median ALEXA global traffic rank was 62,375 with a minimum of five and a maximum of 4,710,605.

Neither the JAMA benchmark criteria, the HON code certification, the ALEXA traffic rank, the country of origin (except slightly superior DISCERN score for UK websites compared to USA websites, *p* < 0.033) nor the ranking within the search results showed a significant association with the respective DISCERN Plus score.

Of all analyzed websites, the vast majority had a clear focus on radiotherapy for prostate cancer; four websites provided only information for radiotherapy in general. All analyzed websites had a focus on curative teletherapy (i.e. definitive radiotherapy to the prostate or to prostatic bed in a postoperative or salvage setting), 76, 51 and 22% of all websites mentioned brachytherapy, active surveillance and palliative radiotherapy (i.e. radiotherapy to other sites than the prostate or prostatic bed, e.g. bone metastases), respectively. In 57% the procedure of radiotherapy starting from first preparation, daily treatment and aftercare was described in detail. Special radiation techniques like “hypo-fractionation”, “Intensity modulated Radiotherapy (IMRT)”, “Image guided Radiotherapy (IGRT)” and “proton therapy” were mentioned in 37, 72, 27 and 31% of all analyzed websites, respectively.

## Discussion

In recent years, the Internet has gained importance as a source for health-related information. Several study groups confirmed this fact in patients with oncological diseases in general [9, 23], as well as for patients with gynecologic cancers [24], breast cancer [19, 20], colorectal cancer [25], bladder cancer [26], laryngeal cancer [27] and prostate cancer [12] in particular. The increasing amount of data published within the last two years highlights its clinical relevance.

For prostate cancer in particular there is a high need for information because several treatment strategies exist. Radiotherapy represents a major component for the management of this oncologic entity. Thus, we performed an evaluation of websites addressing information on prostate cancer and radiotherapy. The first 20 websites were subjected to further evaluation as it is known that patients rarely browse through further sites when searching the web [13]. Ngyuen et al. showed that 72% of the patients even restricted their search to 1–5 websites only [14]. In a study of Borgmann et al. the list of evaluated websites was limited to 10 sites [12].

Overall, we found a good quality of information on analyzed websites according to the validated DISCERN Plus score (for Google search: mean DISCERN Plus score: 55.1 ± 10.0). Our results are in concordance with findings from studies analyzing website quality of other cancer entities, returning a mean DISCERN Plus score of 50 [27], 52 [25], 42 [28] and 42 [20], respectively. Notwithstanding, the only other comparable study evaluating websites with general information on prostate cancer, obtained higher DISCERN Plus scores (mean 77) [12]. Taken together, websites with information on prostate cancer seem to be of slightly better quality compared to websites covering other cancer entities. A potential reason why the web search by Borgmann et al. returned webpages of higher quality might be associated with the use of a more general and popular search term. This is reflected by a lower ALEXA traffic rank for prostate cancer sites in general (median ALEXA traffic rank in the study of Borgmann et al.: 2718) compared to our focus on radiotherapy and prostate cancer (median ALEXA traffic rank: 62375). In our study, webpage quality (DISCERN Plus score 2.14 ± 1.03) of less frequently visited websites (*n* = 14, ALEXA traffic rank < 62,375) was not significantly different from more frequently visited sites (*n* = 15, ALEXA traffic rank > 62,375, DISCERN Plus score 1.67 ± 0.98). We did not find an association of ALEXA traffic rank and superior DISCERN score (data not shown).

In our analysis only 13% of websites were HON code certified. Most other studies evaluating websites on cancer entities in general found higher certification rates ranging from 20 to 38% [12, 19, 20, 26, 29–31]. The vast majority of these studies showed that a higher HON code certification was not necessarily accompanied by a better content quality, as determined by the DISCERN Plus score. Borgmann et al. presumed a lower HON code certification frequency for websites on more specific topics, e.g. prostate cancer and radical prostatectomy. This phenomenon could also account for the low number of HON code certified websites in our study, which is in concordance with findings by the study group of Alkhateeb indicating 17% of HON code certification in their focused web search on prostate cancer and surgery [32].

The websites operated by charity organizations were of superior quality concerning the DISCERN score compared to hospital sites and sponsored medical news sites. In line with this finding, Liebl et al. also found websites of non-profit providers and self-help groups of superior quality compared with profit-driven websites for cancer patients in general [33]. Moreover, an earlier analysis of our study group also revealed better results for hospital and NGO websites compared to sponsored medical news sites for webpages with information on breast cancer [20]. Thus, currently non-commercial sites should be preferentially recommended to patients seeking information on prostate cancer and radiotherapy.

No significant differences in the DISCERN Plus score, JAMA benchmark criteria and HON code certification were found between the three search engines (data not shown). Search results obtained via Google were greatly directed at laypersons and showed a high grade of consistency (i.e. exclusion rates, duplicate websites and temporal changes in search rank), in contrast to the other search engines. These properties might render Google a reliable choice for laypersons searching for information on prostate cancer and radiotherapy.

Interestingly, a large percentage of analyzed websites provided information on modern radiotherapy techniques like IMRT (72%). Also, websites mentioned brachytherapy in the setting of curative treatment in 76% and the possibility of no treatment (active surveillance) in 51%. Despite the well-balanced information on modern treatment options for localized disease, we found a significant lack of information on palliative radiotherapy, which was only mentioned by 22% of websites.

### Limitations

One has to be aware that besides the influence of temporal changes, internet search results may vary based on e.g. the choice of search engine, search term, search date and country of origin settings within the search engine. However, within Google.com, no substantial differences in search results were observed using different search terms (e.g. “radiation” or “radiation therapy” instead of “radiotherapy”) or countries of origin (data not shown).

Websites with complete access restricted by password, websites with personal experiences only (e.g. blogs, videos) or with limited information on radiotherapy (< one paragraph), newspaper articles and PowerPoint presentations were excluded from further analysis. We are aware of the fact that those sources may also contain meaningful information for prostate cancer patients. However, the exclusion was executed to apply the above-mentioned tools in order to standardize evaluation and to preserve comparability. This was handled similar in other publications [15, 16].

## Conclusion

Websites on radiotherapy and prostate cancer directed at laypersons have the potential to sufficiently inform patients about general treatment options. The fact that we were unable to find a simple strategy for the identification of high-quality websites (i.e. HON code certification, JAMA benchmark criteria, ALEXA ranking, different search engines or country of origin and DISCERN Plus score) emphasizes the responsibility of the treating physicians to interpret and rank the vast quantity of information.

## Additional file

Additional file 1: web domains of the first google search (DOC 28 kb)

## Abbreviation

HON: Health On the Net Foundation.
IMRT: Intensity modulated Radiotherapy.
IGRTA: Image guided Radiotherapy.
JAMA: Journal of the American Medical Association.
NGO: Non-governmental organizations.
SD: Standard deviation.
