# Supplementary material for: Radiotherapy for prostate cancer: DISCERN quality assessment of patient-oriented websites in 2018
Source: BMC Urol. 2019 May 28;19:42. doi: 10.1186/s12894-019-0474-4 (PMC6537434; doi:10.1186/s12894-019-0474-4)
Supplement: Supplementary file 1 — web domains of the first google search (DOC 28 kb) [file 12894_2019_474_MOESM1_ESM.doc]

Google search august 22nd 2017

1. <http://www.cancerresearchuk.org/about-cancer/prostate-cancer/treatment/radiotherapy-treatment>
2. <https://prostatecanceruk.org/prostate-information/treatments/radiotherapy-for-advanced-prostate-cancer>
3. <https://www.cancer.org/cancer/prostate-cancer/treating/radiation-therapy.html>
4. <http://www.macmillan.org.uk/information-and-support/prostate-cancer/early-prostate-cancer/treating/radiotherapy/radiotherapy-explained/radiotherapy-for-early-prostate-cancer.html>
5. <https://www.targetingcancer.com.au/2015/02/radiation-therapy-95-effective-prostate-cancer/>
6. <https://www.theprostatecentre.com/prostate-information/treatment-for-prostate-cancer-that-has-not-spread/radiotherapy/>
7. <https://www.pcf.org/c/treatment-for-prostate-cancer-external-beam-radiation-therapy/>
8. [http://www.webmd.com/prostate-cancer/guide/prostate-cancer-radiation-therapy#1](http://www.webmd.com/prostate-cancer/guide/prostate-cancer-radiation-therapy#1)
9. <http://urology.ucla.edu/radiation-therapy-prostate-cancer>
10. <https://www.cancercouncil.com.au/prostate-cancer/management-treatment/radiotherapy/>
11. <http://www.mayoclinic.org/tests-procedures/external-beam-radiation-for-prostate-cancer/home/ovc-20204694>
12. <https://www.radiologyinfo.org/en/info.cfm?pg=pros_cancer>
13. <http://www.roboticoncology.com/radiation-therapy-faqs/>
14. <https://zerocancer.org/learn/current-patients/types-of-treatment/r/>
15. <http://www.prostate.org.au/awareness/for-recently-diagnosed-men-and-their-families/localised-prostate-cancer/side-effects/radiotherapy-what-are-the-side-effects-and-ways-of-managing-them/>
16. <http://www.cancercenter.com/prostate-cancer/radiation-therapy/>
17. <https://www.mskcc.org/cancer-care/types/prostate/treatment/radiation-therapy>
18. <http://prostatecancer.ca/Prostate-Cancer/Treatment/Radiation-Therapy>
19. <http://www.urologyhealth.org/urologic-conditions/prostate-cancer/treatment/radiation-therapy>
20. <http://www.cancer.ca/en/cancer-information/cancer-type/prostate/treatment/radiation-therapy/?region=on>
